# A Smartphone-Fluidic Digital Imaging Analysis System for Pancreatic Islet Mass Quantification

**DOI:** 10.3389/fbioe.2021.692686

**Published:** 2021-07-19

**Authors:** Xiaoyu Yu, Pu Zhang, Yi He, Emily Lin, Huiwang Ai, Melur K. Ramasubramanian, Yong Wang, Yuan Xing, José Oberholzer

**Affiliations:** ^1^ Department of Surgery, University of Virginia, Charlottesville, VA, United States; ^2^ Department of Mechanical and Aerospace Engineering, University of Virginia, Charlottesville, VA, United States; ^3^ Department of Molecular Physiology and Biological Physics, University of Virginia, Charlottesville, VA, United States

**Keywords:** smartphone, microfluidic, video processing, human islets transplantation, diabetes, islet equivalent

## Abstract

Islet beta-cell viability, function, and mass are three decisive attributes that determine the efficacy of human islet transplantation for type 1 diabetes mellitus (T1DM) patients. Islet mass is commonly assessed manually, which often leads to error and bias. Digital imaging analysis (DIA) system has shown its potential as an alternative, but it has some associated limitations. In this study, a Smartphone-Fluidic Digital Imaging Analysis (SFDIA) System, which incorporates microfluidic techniques and Python-based video processing software, was developed for islet mass assessment. We quantified islets by tracking multiple moving islets in a microfluidic channel using the SFDIA system, and we achieved a relatively consistent result. The counts from the SFDIA and manual counting showed an average difference of 2.91 ± 1.50%. Furthermore, our software can analyze and extract key human islet mass parameters, including quantity, size, volume, IEq, morphology, and purity, which are not fully obtainable from traditional manual counting methods. Using SFDIA on a representative islet sample, we measured an average diameter of 99.88 ± 53.91 µm, an average circularity of 0.591 ± 0.133, and an average solidity of 0.853 ± 0.107. *Via* analysis of dithizone-stained islets using SFDIA, we found that a higher islet tissue percentage is associated with top-layer islets as opposed to middle-layer islets (0.735 ± 0.213 and 0.576 ± 0.223, respectively). Our results indicate that the SFDIA system can potentially be used as a multi-parameter islet mass assay that is superior in accuracy and consistency, when compared to conventional manual techniques.

## Introduction

Type 1 diabetes mellitus (T1DM) is characterized by the autoimmune destruction of insulin-producing beta-cells within pancreatic islets. Transplantation of pancreatic islets isolated from donated, cadaveric organs can restore normal blood glucose homeostasis. Next-generation approaches with stem-cell derived islets are currently being tested in clinical trials. Islets for therapeutic application are defined as a “biological drug” by the Food and Drug Administration (FDA) and, as such, must meet product release criteria such as purity, viability, mass, and functionality (Hering et al., 2016; Ricordi et al., 2016).

Islet mass is an important parameter that determines transplant outcomes (Shapiro et al., 2000; Gangemi et al., 2008; Wang et al., 2013; Qi et al., 2014). Islet mass quantification is often performed manually under a microscope after dithizone (DTZ) staining of zinc ions in beta-cells, which helps to differentiate islet and acinar tissues. Islet mass is then calculated in terms of islet equivalents (IEq). One IEq is defined as an islet with a diameter of 150 µm. Islets are classified algorithmically into groups by size using 50 µm diameter increments. A final IEq is calculated for each group using relative conversion factors (Buchwald et al., 2009). The manual method has been widely accepted for practice; however, it is highly subjective with significant inter-and intra-operator variability (Navarro-Fontestad et al., 2012).

Several computer-assisted digital imaging analysis (DIA) systems have been implemented which show reduced variability as compared to manual counting (Niclauss et al., 2008; Gmyr et al., 2015; Buchwald et al., 2016; Wang and Kaufman, 2016). However, several shortcomings of DIA still exist. For example, islets often aggregate with or overlay each other during sample preparation, resulting in difficulties in isolating individual islets. Additionally, static 2D-based DIA methods provide limited information on purity, morphology, and cell volume. Importantly, these methods still require operator intervention, leading to human errors and biases that may not meet FDA requirements of Current Good Manufacturing Practice (cGMP).

As an alternative to existing methods of islet quantification, we may look to unique, innovative solutions involving advanced technologies. For instance, smartphones equipped with high resolution complementary metal–oxide–semiconductor (CMOS) cameras, high-performance processing units, and tailored software can be used as analytical devices for biological research and clinical diagnostics (Hutchison et al., 2015). Assisted by advancements in molecular analysis, biosensors, mathematical algorithms, microfabrication, 3D-printing, and microfluidics, smartphones have been used as portable, versatile, and highly-connected read-out platforms capable of capturing the microscopic world ranging from tissues and cells to individual DNA molecules (Lee et al., 2011; Gmyr et al., 2015; McCracken and Yoon, 2016). The imaging capability of smartphones can be extended to the function of a microscope by adding an external lens, allowing for the capture of digital images and photographs with high resolution. Furthermore, with advances in wireless communication and with the help of cloud computing, complex analytical processing can be performed after data acquisition to generate diagnostic results (Im et al., 2015). Compared to conventional laboratory microscopes, smartphone technology provides a more rapid, portable, user-friendly, and cost-effective way to allow even minimally trained users to operate the system in the field.

Another helpful technology is microfluidics, which involves the precise manipulation of fluids at a microscopic scale. Microfluidics offers unique advantages for studying pancreatic islets by closely mimicking the physiological microenvironment. It also allows for the control of stimulation cues and integration of various analytical tools. Since the early 2000s, the development of *in vitro* microfluidic-based tools for diabetes research has drawn significant attention. Such microfluidic devices possess many advantages over more established *in vitro* assays, including reduced reagent consumption requirements, low cost of manufacturing and maintenance, multiplexing capabilities, increased assay sensitivity, increased accuracy, and higher spatiotemporal resolution (Kappler et al., 2004; Adewola et al., 2010; Nourmohammadzadeh et al., 2016; Xing et al., 2016; Lu et al., 2018). An exciting development in recent years is the adaptation of smartphone technology to microfluidic platforms for point-of-care (POC) chemical and biological detection, as well as for particle counting and analysis (Long et al., 2017; Huang X. et al., 2018; Liu et al., 2019; Hassan and Zhang, 2020).

In this paper, we describe a smartphone-based microfluidic system for human islet mass quantification that can provide more comprehensive characterization of isolated human islets. This system may be adopted in the future as a product release method for diabetes cell therapies.

## Materials and Methods

### Design and Fabrication of Smartphone-Fluidics-Based Flow Cytometry

**FIGURE 1:**
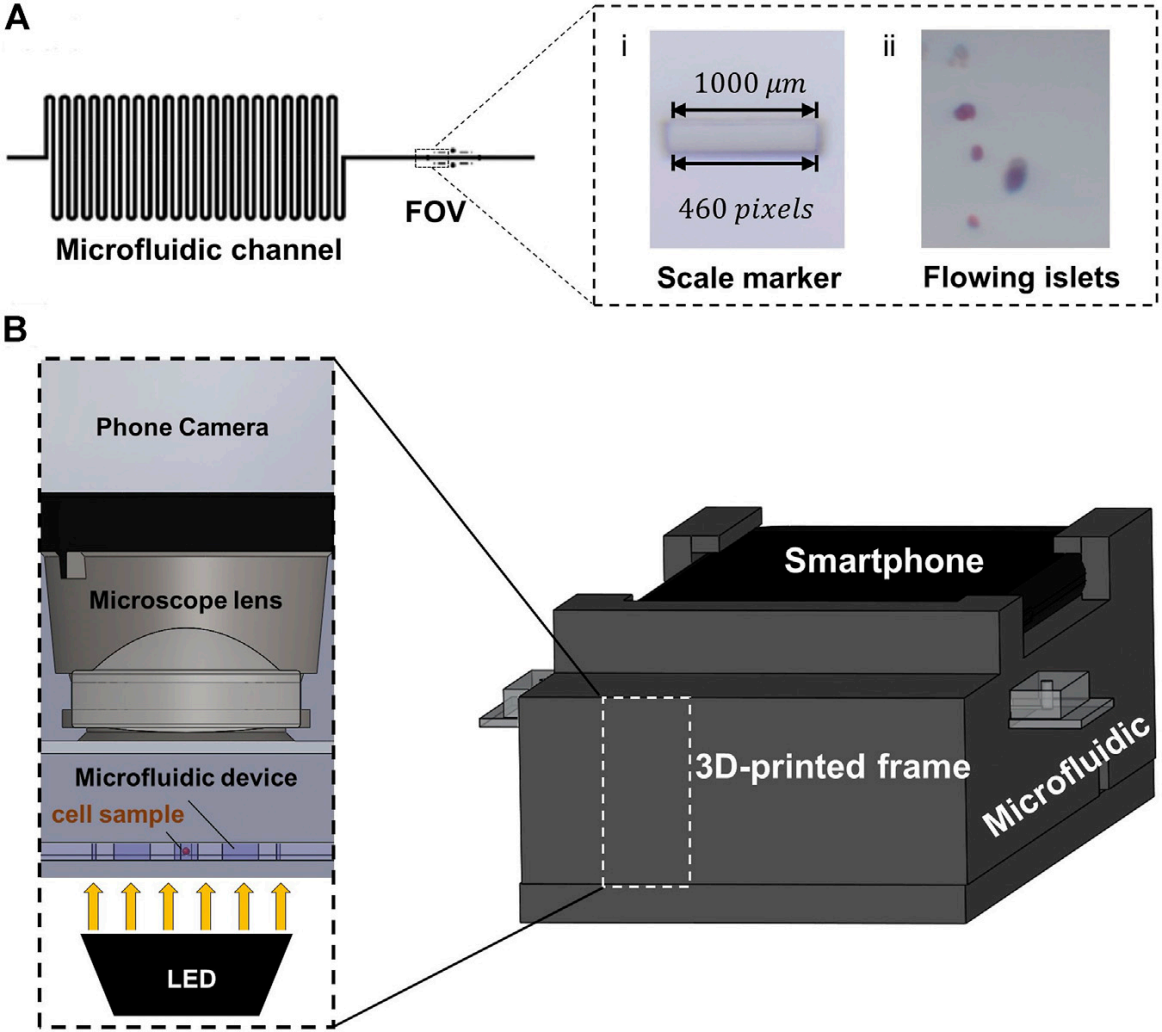
**(A)** Design of the microfluidic device. **(i)** The 1,000 µm marker used to calculate scaling factor δ. **(ii)** An example of video captured by the smartphone camera. **(B)** Hardware design for the smartphone-fluidics-based flow cytometry system. The 3D frame is designed to hold the smartphone at the top, the microfluidic device in the middle, and the LED light source at the bottom.

The Smartphone-Fluidic Digital Imaging Analysis (SFDIA) system consists primarily of two components: a microfluidic device and a smartphone system. The smartphone system includes a 3D printed frame, a magnifying lens (f = 4.51 mm), a light source (Adafruit Industries, New York, NY), and a smartphone (Google Pixel 3, Foxconn, New Taipei, Taiwan) (Figure 1). The microchannel (500 μm in diameter and 500 μm in height) is made of one layer of PDMS (Polydimethylsiloxane, Fisher Scientific, Ontario, CA) using soft-photolithography (Adewola et al., 2010; Nourmohammadzadeh et al., 2016; Xing et al., 2016). This microchannel is bonded to a glass slide (Fisher Scientific, Ontario, CA) (Figure 1A), which is divided into an islet loading area with a repeated loop channel and a straight channel as a viewing area. The repeated loop channel has a total length of 700 mm and a total liquid volume of 250 μl. It is designed to preload islets, to act as a buffer zone for focusing islets in the middle of the channel, and to separate islets in distance. The straight channel is also 700 mm in length and contains a viewing area of 2 mm^2^ for smartphone imaging and video recording. In the Field of View (FOV), there are two embedded scale markers of 1 and 0.1 mm (Figure 1A). These markers serve as a conversion scale calibrated to equivalent pixel density.

The smartphone frame is 3D-printed from polylactide resin (MakerBot® PLA resin, MakerBot® Industries, New York, NY) using a MakerBot® 3D printer (MakerBot® Industries, New York, NY). The frame is designed to hold the smartphone, the microfluidic device, and the optics system (magnifying lens and LED light source) (Figure 1B). The frame is 80 mm in width and 199.3 mm in length. There is a groove channel (20.50 mm in width and 3.21 mm in height) for holding fluids. Between the microfluidic channel and smartphone camera, there is a magnifying lens (A230, Thorlabs, Newton, NJ) held in a socket (6.00 mm in diameter). The lens has a diameter of 6.34 mm with a focal length (f) of 4.51 mm and a numerical aperture (NA) of 0.55. As illustrated in Figure 1B, an LED powered by a 3.0 V lithium battery serves as the illumination source and is placed at the bottom. The LED provides a consistent diffused light to optimize video acquisition. Videos of flowing islets are recorded at a rate of 30 fps by a CMOS camera on the Google Pixel 3 (12.2 MP, f/2.0). The camera is placed directly on top of the magnifying lens with an effective FOV of 2 mm^2^.

### Video Processing Algorithm

**FIGURE 2:**
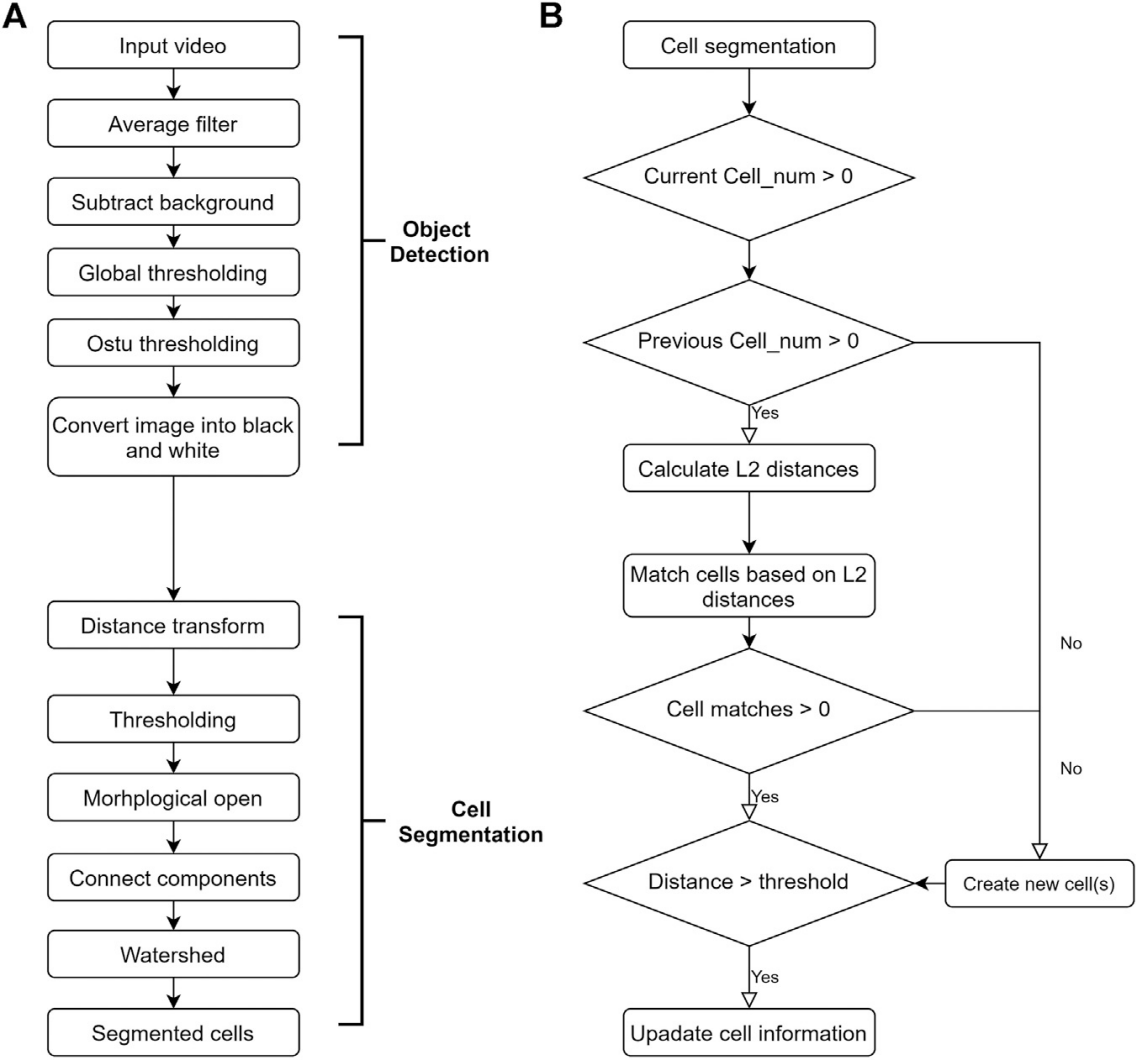
Flow chart of video processing algorithm. **(A)** Flow chart of the individual frame analysis. This can be further divided into object detection and cell segmentation. **(B)** Flow chart of the cell matching algorithm.

The first step in video processing is individual frame analysis. This step mainly consists of two parts: object detection and cell segmentation, as outlined in Figure 2. External environmental factors (lighting conditions, the specific microfluidic device used, etc.) are consistent when videos are taken. As such, background subtraction is a good way to separate background from foreground.

**FIGURE 3:**
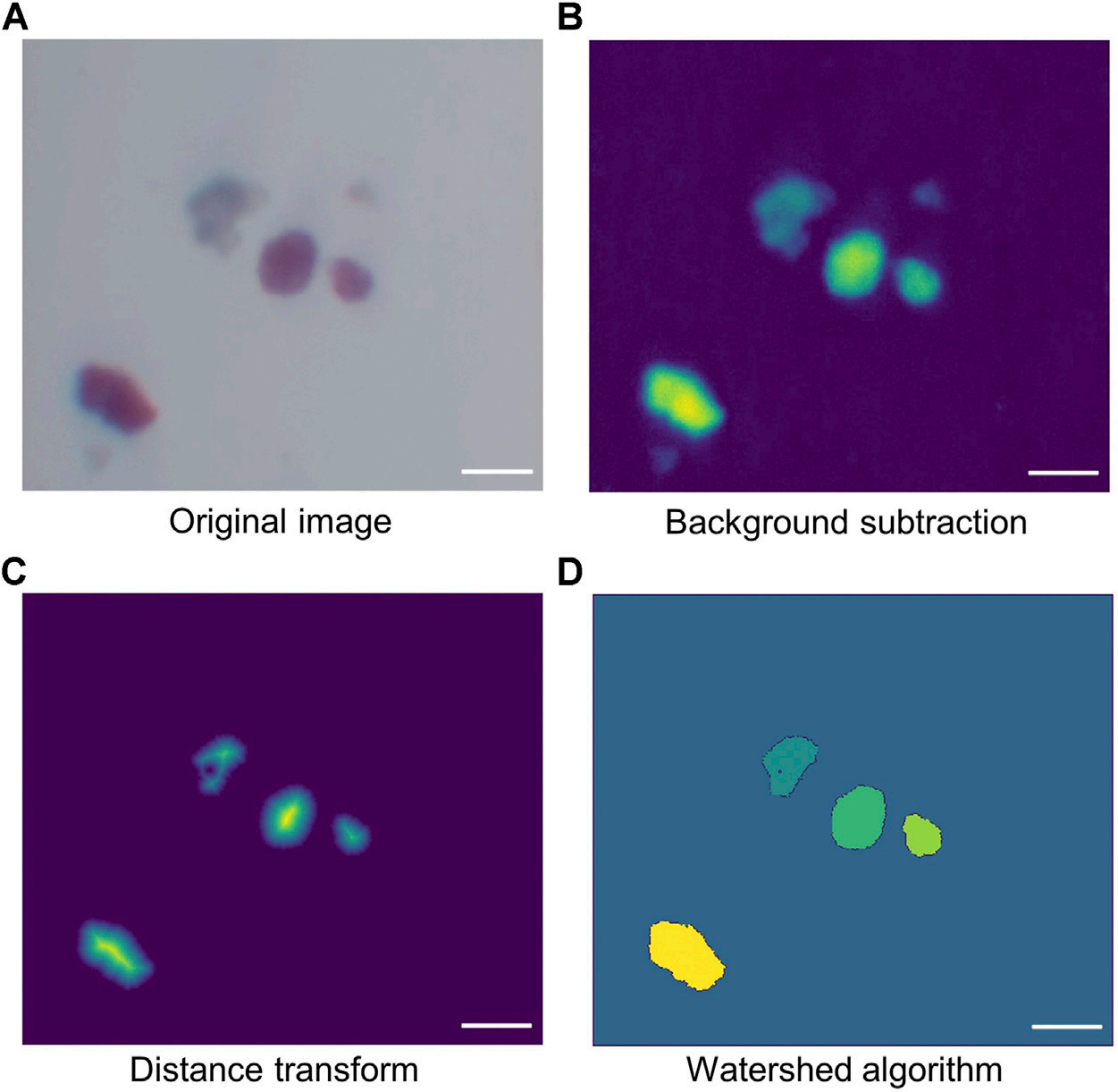
Demonstration of cell segmentation. **(A)** Original image directly obtained from the smartphone camera. **(B)** Image created by subtracting background from the original image. **(C)** Image after applying distance transform. Since distance transform calculates the distance of each pixel from its nearest boundary, the central pixels have higher values. As a result, when applying the proper threshold to the transformed image, small-sized cells as well as peripheral areas of large cells (mainly connections between cells) can be removed, leaving only the central area of large cells. **(D)** Image created by watershed algorithm. Scale bar = 200 µm.

Figure 3 shows the typical processing sequence of a sample video frame. First, a background image is taken. The original islet image is captured directly by the smartphone camera (Figure 3A). Both the actual input video and the background image are then smoothed by a low pass filter. The foreground is obtained by subtracting the background from frames in the input video (Figure 3B). Finally, the foreground is converted into a black and white image using a modified version of Otsu thresholding.

The black and white image is further processed with a distance transform, converting binary foreground pixel values into distances between themselves and the nearest background pixel. As a result, the pixels around the center of each blob contain higher values, while the pixels at the edge of the blob contain lower values. Another threshold is then applied to the transformed image followed by morphological opening. These remove unwanted connections between cells small particles which are considered to be fragments of tissues instead of islets. The resulting image shows only the major, central area of the cells, with unwanted particles removed (Figure 3C). By changing the threshold, the size of the small particles to be filtered out can be controlled. Finally, Watershed algorithm was used to group the non-central foreground pixels based on the cell center clusters made in the previous step. The result is shown in Figure 3D, with cells marked out with different colors.

After analysis of each individual frame, islet matching is performed to ensure that every islet is counted only once. As is outlined in Figure 2B, islet tracking is done using a Euclidean-distance-based approach (Chowdhury et al., 2010). A feature vector is formed for each detected islet. The vector includes the x-coordinate, y-coordinate, and size of the islet. Between two consecutive frames, the weighted Euclidean distance (known as $L_{2}$ distance) J between each cell pair is calculated using the following equation:

$$J=1.0\times \frac{{\left||x_{1}-x_{2}|\right|}_{L_{2}}}{min\left(r_{1},\;r_{2}\right)}+0.5\times \frac{{\left||y_{1}-y_{2}|\right|}_{L_{2}}}{min\left(r_{1},\;r_{2}\right)}+1.0\times \frac{{\left||a_{1}-a_{2}|\right|}_{L_{2}}}{min\left(a_{1},\;a_{2}\right)}$$ (1)

where $x_{1}$ and $x_{2}$ are the x-coordinates, $y_{1}$ and $y_{2}$ are the y-coordinates, $r_{1}$ and $r_{2}$ are the radii of the two cells, and $a_{1}$ and $a_{2}$ are the areas. The weights (1, 0.5, 1) are determined based on the fluid flow rate (100 μl/min) and video frame rate (30 fps). Individual islet clusters captured from two consecutive frames are matched based on shortest distance. *Via* further testing, we have shown that these weights can distinguish between matched and unmatched cells.

### Quantification of Islet Mass

#### Size, Volume, and Islet Equivalents Quantification

Equivalent spherical diameter (ESD) has been widely used for size quantification of irregularly shaped particles (Jennings and Parslow, 1988). In this study, the equivalent spherical diameter of an individual islet is defined by the following equations:

$$D_{pixel}=2\sqrt{\frac{A_{pixel}}{\text{π}}}$$ (2)

$$D_{\mu m}=D_{pixel}\times \delta$$ (3)

where $D_{pixel}$ represents the islet’s equivalent diameter in pixels and $A_{pixel}$ represents the islet’s area in pixels. $D_{pixel}$ is converted to diameter in micrometers ($D_{\mu m}$) using a conversion factor δ, which is calculated by measuring a built-in marker (1,000 µm) as is shown in Figure 1A. The calculation of δ is shown in Eq. 4. The length of the 1000 µm marker was measured to be 460 pixels.

$$\delta =\frac{1000\mu m}{Length_{pixel\;of\;1000\mu m\;markers}}$$ (4)

IEq assessment is done, as previously established, by classifying $D_{pixel}$ into eight size ranges in μm (50–100, 101–150, 151–200, 201–250, 251–300, 301–350, 351–400, and >400). Then, IEq can be calculated by multiplying the number of islets in each group with corresponding multipliers (0.167, 0.667, 1.685, 3.499, 6.315, 10.352, 15.833, and 22.750, respectively) (Ricordi et al., 1990).

**FIGURE 4:**
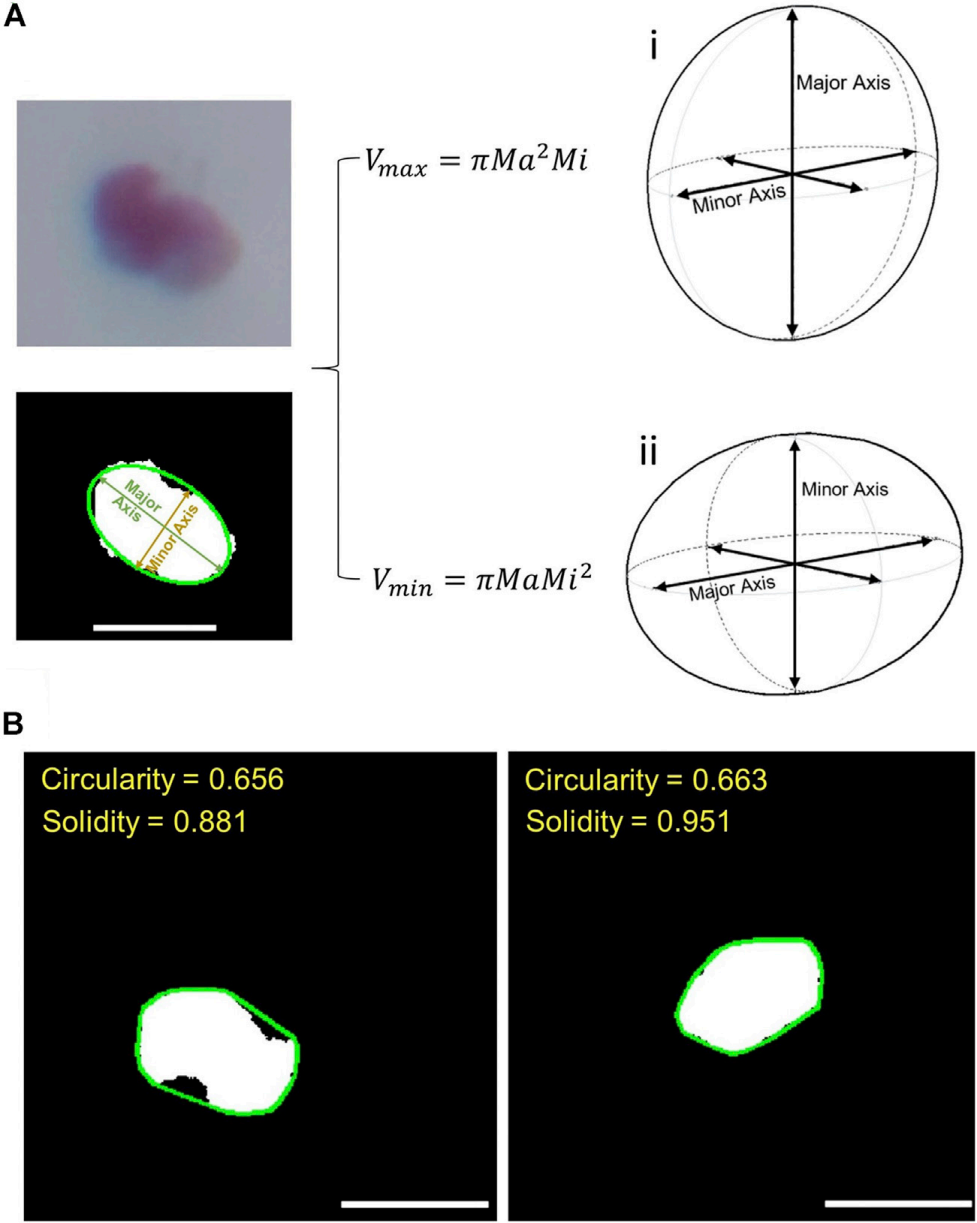
**(A)** Demonstration of cell volume estimation. The two figures on the left show the process of ellipse fitting. The two figures on the right show the two different methods of estimating volume after ellipse fitting: **(i)** the calculation of ellipsoid volume when the third axis is the major axis; **(ii)** the same calculation when the third axis is the minor axis. **(B)** Demonstration of circularity and solidity assessment. The green lines in the two images represent the convex hull for the two islets. Solidity is defined as the ratio between actual cell size (white area) and the area surrounded by the convex hull. Scale bar = 200 µm.

Under most circumstances, islets have irregular shapes. As a result, in 2-D images, an ellipse generally better represents the shape of an islet than a perfect circle (Girman et al., 2003). Thus, volume estimation is performed based on a 3-D Ellipsoid-Fitting-based algorithm. As demonstrated in Figure 4A, each islet is first fitted to an ellipse using least-squared approximation (Fitzgibbon et al., 1999) using a function provided by OpenCV™. Lengths of the major and minor axes were obtained. The 2-D ellipse can be converted to a 3-D ellipse by revolving the ellipse around the minor axis and using the major axis of the 2-D ellipse as the third axis of the ellipsoid as shown in Figure 4Ai (Girman et al., 2003). The volumes of islets in each frame are estimated using Eq. 5:

$$V_{pixel}=\frac{4}{3}\pi Ma^{2}Mi$$ (5)

where Ma and Mi represent the long axis and short axis, respectively. The final volume is the average volume across each frame as is defined in Eq. 6:

$$\bar{V}=\frac{{\sum V}_{pixel}}{n}$$ (6)

$V_{pixel}$ represents the estimated volume of a cell in a single frame using pixels, and ${\bar{V}}_{pixel}\;$represents the average estimated volume of a cell using pixels. ${\bar{V}}_{pixel}$ can be then converted to ${\bar{V}}_{\mu m^{3}}$ using the scaling factor δ as shown in Eq. 7.

$$\bar{V_{\mu m^{3}}}=\bar{V_{pixel}}\times \delta^{3}$$ (7)

The Ellipsoid-Fitting-Based Volume (EFV) was compared to the volume obtained based on IEq assessment (IEqV). According to the definition of IEq, IEqV can be calculated using the following equation:

$$IEqV=IEq\times V_{150\mu m}$$ (8)

where IEq is the Islet Equivalent obtained from the previous step and $V_{150_{\mu m}}$ is the volume of islets with a diameter of 150 μm, which is calculated to be 1.767 × 10^6^ μm^3^ using the sphere volume equation:

$$V=\frac{4}{3}\pi r^{3}$$ (9)

When estimating ellipsoid volume based on 2-D ellipse information, this work initially used Eq. 5 as discussed earlier. Yet there is another way of such estimation, which is to revolve a 2-D ellipse around its major axis, and use the minor axis as the third axis of the ellipsoid as shown in Figure 4Aii (Niclauss et al., 2008) resulting in Eq. 10:

$$V_{pixel}=\frac{4}{3}\pi MaMi^{2}$$ (10)

In this paper, both methods were implemented in order to see whether the choice of a different ellipsoid calculation method would have a significant impact on overall islet volume assessment.

#### Circularity and Solidity Quantification

A spherical model is generally used in the 3D representation of islets. Roundness (circularity) is used to measure the level of an islet’s shape regularity (Olehnik et al., 2017; Huang H.-H. et al., 2018). Circularity is a number ranging from 0 to 1.0. Higher circularity means that the shape of the given islet is closer to a circle. In this work, average circularity was calculated using the following equations:

$$\bar{C}=\frac{\bar{A_{actual}}}{\bar{A_{estimated}}}$$ (11)

Aestimated¯=∑0nP2/actual(4π)n (12)

where $\bar{C}\;$stands for average circularity, $\bar{A_{actual}}$ stands for actual islet area, $\bar{A_{estimated}}$ stands for average estimated particle area, and $P_{actual}$ stands for actual particle perimeter. $P_{actual}$ is obtained by counting the number of pixels on the islet’s contour, which is then converted to micrometers.

In addition to circularity, we applied the concept of solidity as an indication of islet shape regularity. The solidity is defined as the ratio of the area of the particle’s convex hull to the area of the actual particle:

$$Solidity=\frac{\bar{A_{convexHull}}}{\bar{A_{actual}}}$$ (13)

The convex hull was obtained using a function provided by OpenCV™. Like circularity, solidity is a number ranging from 0 to 1.0. It is demonstrated in Figure 4B, where the green lines represent the convex hulls of the two islets. Since both islets have an elliptical shape, they have very similar circularities; however, the islet on the left is a bit more fragmented than the one on the right, and this difference is reflected in solidity.

### Trapped Islet Percentage Estimation

Trapped islet percentage is estimated using the positive area of DTZ staining of zinc in the islets and computed as:

$$\%DTZ^{+}ratio=\frac{Total\;dithizone\;positive\;area}{Total\;islet\;area}$$ (14)

The positive area normally produces a red color after DTZ staining, which can be identified based on Hue in the HSV (Hue, Saturation, Value) color space in digital image processing. In this work, pixels with a Hue value between 310 and 360 (Hue value is set in a range of 0–360) were identified as zinc positive areas.

**FIGURE 5:**
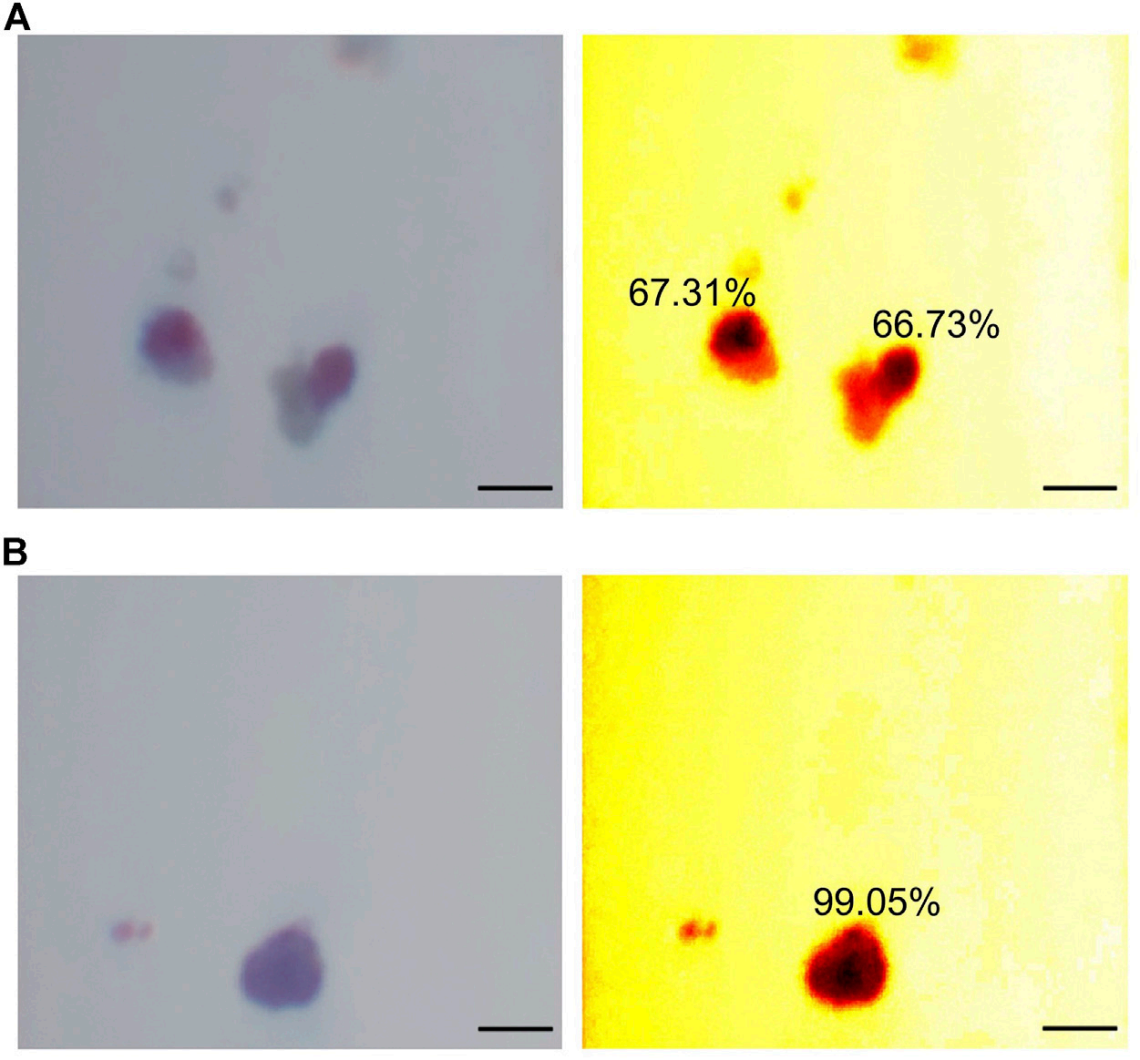
Demonstration of trapped islet percentage. **(A)** Example of two trapped islets. **(B)** Example of a free islet. The two images on the left are raw images taken from the smartphone camera. The two images on the right are heatmaps of HSV color space. Scale bar = 200 µm.

Figure 5 includes example images of trapped islets (Figure 5A) and free islets (Figure 5B). The images on the left were taken directly from the smartphone camera. For better visualization, two heatmaps of HSV color space were generated. In the heatmaps, the DTZ stained areas appeared dark red/black, while the no-DTZ areas were bright red. 50% of the area of the two trapped islets in Figure 5A was stained with DTZ, while the free islets in Figure 5B were almost completely stained with DTZ. The DTZ positive ratios of the two trapped islets were calculated to be 67.31 and 66.73%, while the ratio of the free islets was calculated to be 99.05%.

### Human Islet Preparation and Manual Mass Quantification

Human islets were isolated according to the published protocol (Qi et al., 2009a; b). In brief, a cadaver pancreas was obtained from organ procurement organizations (OPO) and islets were isolated at the University of Virginia with donors’ consent for research. The pancreas was trimmed and distended with Liberase HI (Roche Applied Science, IN) and digested in a Ricordi chamber. The digested tissues were then purified by a continuous UIC-UB gradient protocol on a cell separator (Cobe 2991, Cobe Inc., CO) and then cultured in CMRL 1066 media with 5% human albumin (CSL Behring, King of Prussia, PA) at 37°C under 5% CO_2_.

Dithizone solution (DTZ.,1.5 mM, Sigma-Aldrich, MO) was used to differentiate islets and acinar tissues by staining zinc ions, which are highly concentrated in beta-cells (Ricordi et al., 1990). Tissues were incubated at 37°C for 10 min and then washed twice with PBS. Manual assessment of human islets was done under a microscope in a Petri dish labeled with measurement scales. Diameters of islets were estimated according to these scales. These diameters were used to classify islets into eight size ranges and then converted into IEq with the corresponding multipliers as described earlier in *Size, Volume, and Islet Equivalents Quantification*.

### Human Islet Loading and Video Recording on-Chip

The counting samples of isolated human islets were picked up manually and randomly *via* polyethylene tubing (Intramedic, PE160) connected to a 1 ml syringe. The tubing was then connected to the inlet of the microfluidic channel. The islets were injected into the device *via* PHD ULTRA™ Syringe Pump (Harvard Apparatus, MA) at a flow rate of 20–30 μl/min.

The captured videos (1920 × 1,080; 1080p, 60 fps) were recorded in mp4 file format and then transferred to a computer for further video processing. The video processing algorithm was developed using python 3.8.1 within Spyder IDE (version 3.36). Video analysis was assembled for five functions: object detection, cell segmentation, cell tracking, feature extraction, and report generation. Figure 2 summarizes the procedures of object detection, cell segmentation, and cell tracking.

### Statistical Analysis

Data are expressed as mean ± SD. For the processing of flowing islet videos, *n* = 5 experiments were conducted for each islet sample. Both manual and smartphone-based assessments were performed. Statistical significance was calculated using t-tests (*p*-value < 0.05).

## Results and Discussion

### Human Islet Quantification

**FIGURE 6:**
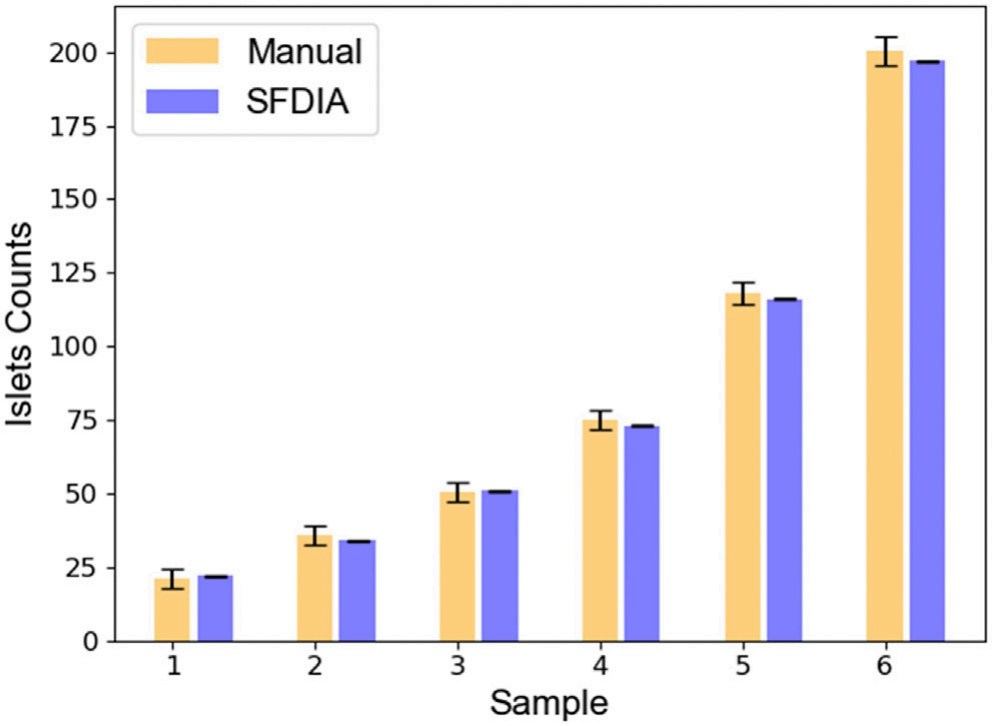
Comparison between manual counting and smartphone-based counting. Six human islet samples of varied sample sizes were assessed by both manual counting and the SFDIA system. Results were plotted and expressed in mean +/− SD. NS: Not Significant (*p*-value > 0.05), *N* = 5.

In the current practice of human islet isolation, about 500 µl of cell suspension, which normally contains 0.05–0.1% of the total islet population, is picked up manually, randomly, and repeatedly for islet mass quantification (Qi et al., 2009a; b). The randomness of sample sizes often causes variation in assessment results. In this study, human islet samples with different islet masses were quantified by both manual counting and the SFDIA system for comparison. Six groups of samples were tested. Islet numbers within each sample ranged from 20 to 200. Experimental results were plotted as shown in Figure 6. Across the six groups, the average difference between the SFDIA system and manual counting was 2.91 ± 1.50%. The *p*-values for all six groups were greater than 0.05, indicating that there is no significant difference between the result generated by manual counting and the SFDIA system. In other words, the SFDIA system yields accurate quantification assessments close to those obtained by manual counting, regardless of islet sample sizes. However, manual counting resulted in a relatively high variation (SD = 3.1–5.0), while the SFDIA system gave consistently low variation (SD < 1). In general, for traditional manual counting, a smaller sample size creates a larger error in the results. The consistency of the SFDIA system successfully reduced human error and rendered the assessment results more reliable than those obtained from the traditional manual counting method.

### Islet Size and Volume Estimation

In addition to cell numbers, we acquired and analyzed multiple parameters pertaining to flowing islets in the microfluidic channel, as discussed in *Quantification of Islet Mass*. Data acquisition and analysis were performed automatically during video processing.

**FIGURE 7:**
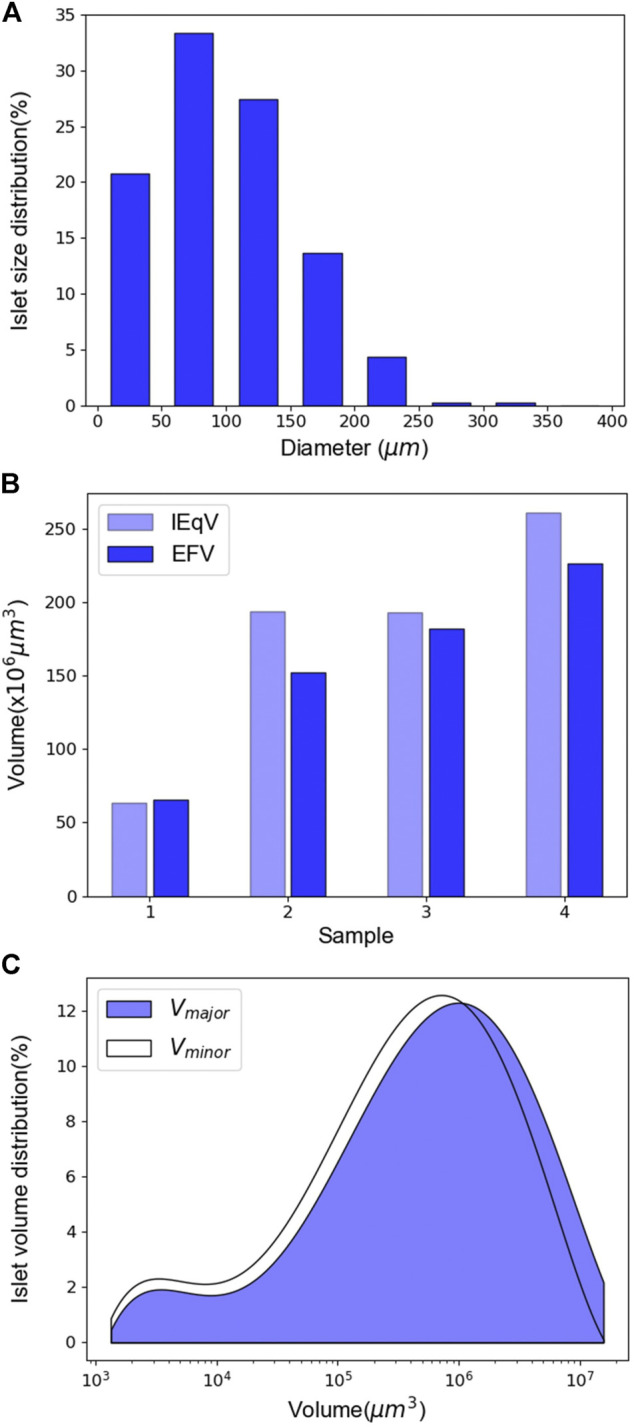
Islet diameter and volume assessment. **(A)** Distribution of islet diameter. The mean diameter of the islet sample is 99.87 ± 53.91 µm. *N* = 628. **(B)** Comparison between IEqV and EFV. *N* = 4. **(C)** Distribution curves of the volume calculated using the two different methods. *N* = 628.

Since islets do not have a perfect regular shape, their diameters were estimated as their equivalent diameters. The equivalent diameter is calculated from islet areas as seen in each video frame. Distribution of human islet diameters are shown in Figure 7A. The islet samples we tested had an average diameter of 99.88 ± 53.91 µm and a maximum diameter of 337.67 µm. The majority (>60%) had diameters between 50 and 150 µm. While the diameter obtained by the SFDIA system is the equivalent diameter as described above, manual assessments often use the length of the major axis as the diameter. As a result, the SFDIA diameter can be slightly smaller but more reliable than that estimated by manual assessment.

We next compared IEqV and EFV. As discussed previously, IEqV is the volume calculated using the conventional IEq estimation method. EFV is calculated using the ellipsoid volume equation described by Eq. 5. As shown in Figure 7B, in samples 2 through 4, IEqV was higher than EFV, while in sample 1, the two had similar values. It is worth noting that sample 2 and 3 had very similar IEqV’s while their EFV’s differed dramatically. This mainly results from the IEqV calculation: islets are first grouped by diameter, and then the number of islets in each group is multiplied by the IEq conversion factor. As a result, IEqV tends to ignore small size variation and leads to greater error.

Two methods of islet volume estimation were compared: V_major_ uses the major axis as the third axis of the ellipse, while V_minor_ uses the minor axis. As shown in Figure 7C, our results indicated that the V_major_ curve was slightly to the right of that of V_minor_ as expected. However, the two curves largely overlapped, indicating that the two methods generated similar results in terms of islet volume estimation. As such, the choice of the third axis in a 2D-ellipse fitting model does not significantly affect islet volume estimation. Our system’s islet volume estimation better represents actual islet volume than traditional IEq-derived islet volume. Thus, our system provides more accurate information to clinicians preparing for islet transplants.

### Islet Fragmentation: Circularity and Solidity Estimation

Islet fragmentation is often caused by chemical and mechanical stress during islet isolation and post-isolation culture (Kin et al., 2008). Fragmentation is a key parameter in the evaluation of islet quality. It has also been considered an important influencer of post-transplant graft survival rates. Therefore, the accurate assessment of human islet fragmentation prior to transplantation is important for improving clinical outcomes.

**FIGURE 8:**
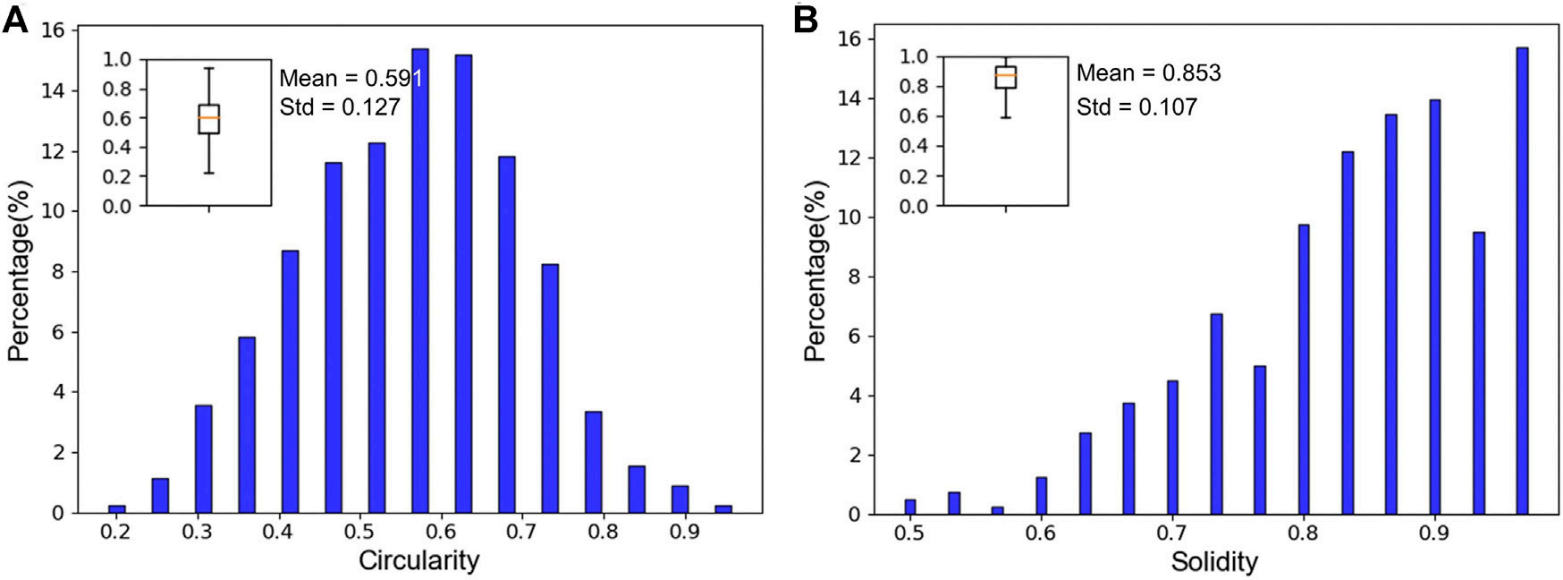
Circularity and solidity assessment. **(A)** Islet circularity distribution. The islet sample has an average circularity of 0.591 ± 0.127. *N* = 628. **(B)** Islet solidity distribution. The islet sample has an average solidity of 0.853 ± 0.107. *N* = 628.

Circularity has been widely used to measure islet fragmentation in islet assessments (Huang H.-H. et al., 2018). An ellipse/ellipsoid model is considered to be a better estimation of islet size and volume than a circular model, since circularity alone may not be enough to quantify shape regularity. In this study, both circularity and solidity were calculated to describe islet morphology. Our human islet samples had an average circularity of 0.591 ± 0.133. As shown in Figure 8A, circularity of the majority of islets (∼83%) ranged between 0.4 and 0.7. As in Figure 8B, the solidity of the samples averaged 0.853 ± 0.107. The majority of islets (∼70%) had solidity values between 0.8 and 1. The high solidity values demonstrate a low rate of fragmentation in our preparation. The low circularity values indicate that the shapes of most islets in our samples are better represented as ellipses than perfect circles. This implies that islet solidity can be used as an improved quantification method for describing islet morphology (roundness, shape, and fragmentation).

### Islet Purity: Trapped Islet Percentage

Despite recent progress in the field of human islet transplantation, low purity of islet products still majorly affects its success rate. Islet cell percentage determines competition for oxygen and other nutrients between islet and non-islet tissues within transplanted allografts. The conventional method to estimate the purity of isolated islets involves DTZ staining and manually counting DTZ-positive islets under a light microscope. The operator-dependent nature of this process and the use of islet count as a unit can lead to significant overestimation of purity (Pisania et al., 2010; Kitzmann et al., 2014). Our software automatically analyzes the trapped islet percentage based on area data acquired from image processing. This provides a more accurate estimation of islet product purity.

**FIGURE 9:**
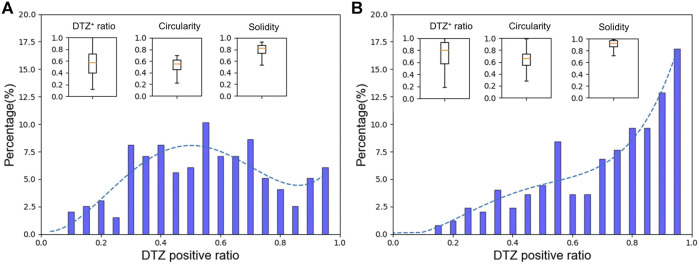
Trapped islet percentage assessment. **(A)** Assessment for middle layer islets. The islet sample has an average DTZ^+^ ratio of 0.576 ± 0.223, an average circularity of 0.534 ± 0.102, and an average solidity of 0.793 ± 0.102. *N* = 198. **(B)** Assessment for top layer islets. The islet sample has an average DTZ^+^ ratio of 0.735 ± 0.213, an average circularity of 0.635 ± 0.139, and an average solidity of 0.853 ± 0.107. *N* = 530.

As shown in Figure 9, we compared the percentage of trapped islets between two groups: middle-layer and top-layer human islets. These two layers generally vary significantly in morphology and tissue composition. In Figure 9A, the average staining ratio of the middle-layer sample was 0.576 ± 0.223. The distribution curve was skewed to the left. ∼72% of islets had DTZ positive ratios between 0.3 and 0.7. These islets had a mean circularity of 0.534 ± 0.102 and a mean solidity of 0.793 ± 0.101. In contrast, Figure 9B shows the result of top-layer islets. The average staining ratio among the top-layer islet samples was 0.735 ± 0.213, with the majority (∼67% of total islets) above 0.7. These islets had a higher mean circularity (0.636 ± 0.139) and a higher mean solidity (0.854 ± 0.107). With our video processing program, we obtained results consistent with expected values from typical islet preparations. We also produced more detailed and reliable information on islet quality than purity estimations from standard manual quantification. Our higher-quality data may prove more useful to clinicians.

## Conclusion

We described a novel and dynamic smartphone-based digital imaging system integrated with microfluidic technology for pancreatic islet mass quantification. Our software program can track multiple moving islets and separate closely attached islets. SFDIA generated little variance in islet counting across multiple experiments, exhibiting improved consistency as compared to the conventional manual counting method. In addition, the system estimated size using an ellipsoid model and returned additional islet parameters such as volume, circularity, solidity, and trapped islet percentage. These parameters are impossible to directly identify with the conventional method. Using our low-cost and portable system, reliable islet parameters can be easily obtained during the preparation of islet biologics.

Several limitations of this study need be mentioned. First, due to the relatively low resolution and frame rate of a smartphone camera, video processing requires a consistent fluid flow. This consistent fluid flow assures a reliable reading of islet parameters but requires an external pump system. To overcome this issue, upgrading to a higher-quality camera is favorable. With the upgrade, it is possible to simplify the system by removing the external power source for fluid delivery. This results in a pumpless microfluidic system as we introduced previously for islet function tests (Xing et al., 2016). Another limitation of our methodology is that our imaging is captured in 2D, but islet mass parameters are truly dependent on the 3D structure of the islets. Although our system is simple and has significant advantages over traditional manual counting, a 3D scanning and modeling approach can further improve accuracy.

In conclusion, manual counting tends to overestimate the islet mass, the volume, and the purity of islet samples due to human errors or method limitations. Through advanced analysis, our SFDIA program analyzes more islet quantification parameters beyond traditional islet mass quantification. As more information becomes accessible *via* our system, we suggest an alternative, multi-parameter islet quality assessment method. This new method would allow operators or physicians to access these islets’ properties objectively and with higher accuracy. Future applications of this smartphone-fluidic system could include a functional potency assay for a comprehensive product release test.

## Data Availability

The raw data supporting the conclusion of this article will be made available by the authors, without undue reservation.
